# Early infant male circumcision for human immunodeficiency virus prevention: knowledge and attitudes of women attending a rural hospital in Swaziland, Southern Africa

**DOI:** 10.1080/17290376.2014.929530

**Published:** 2014-06-24

**Authors:** Prudence Jarrett, Merav Kliner, John Walley

**Affiliations:** ^a^ BSc Student of International Health, University of Leeds, Leeds, UK.; ^b^ BA, MBChB, MPH, MFPH, is a Specialist Registrar in Public Health, University of Leeds, Leeds, UK; ^c^ MBBS, DTMH, MComH, is Professor at the Nuffield Centre for International Health & Development, Leeds Institute of Health Sciences, University of Leeds, Leeds, UK

**Keywords:** circumcision, HIV, sub-Saharan Africa, Circoncision, VIH, Afrique sub-saharienne

## Abstract

Swaziland has the highest prevalence of human immunodeficiency virus (HIV) in the world at 26% of the adult population. Medical male circumcision (MMC) has been shown to reduce the risk of acquiring HIV from heterosexual sex by up to 60% and the Government of Swaziland has been promoting adult male circumcision. Infant circumcision commenced in 2013 so it is important to understand the knowledge and views of women as potential mothers, around infant circumcision for medical purposes to inform the development of the service. This study interviewed 14 women of reproductive age attending the outpatient department of Good Shepherd Mission Hospital (GSMH), a rural district hospital, on their knowledge of and attitudes to early infant male circumcision (EIMC). Participants were highly knowledgeable about the health benefits of medical circumcision, although knowledge of the comparative risks and benefits of EIMC to adult circumcision was poor. All participants would have a son circumcised; the preferred age varied from early infancy to adolescence. Complications and pain were the main barriers whilst religious and cultural reasons were mentioned both for and against circumcision. A variety of family members are important in the decision to circumcise a young boy. Acceptability of medical circumcision was high in this study, but concerns about safety, pain, autonomy and cultural factors reduce the acceptability of infant circumcision more specifically. It will be important to provide accurate, culturally sensitive information about infant circumcision to mothers, fathers and grandparents using existing hospital and community services provided at GSMH and throughout Swaziland. Where possible services for MMC should be available to males of all ages so that families and young men may choose the most favourable age for circumcision.

## Résumé

Le Swaziland a la fréquence la plus élevée du VIH du monde avec 26% de la population adulte touchée. On a montré que la circoncision peut réduire le risque de contracter le VIH des rapports hétérosexuelles de jusqu’à 60%, et le gouvernement du Swaziland promeut la circoncision médicale masculine. Le programme de la circoncision d'enfants commencera en 2013. Vu que la circoncision d'enfants est imminent, il est ainsi important d’évaluer les connaissances et les attitudes de femmes en tant que mères, au niveau de la circoncision afin d'informer le développement du service. Au cours de cette étude, des entretiens ont été menés avec quatorze jeunes femmes de l’âge reproductif qui allaient au service des consultations externes l'hopital de la mission Good Shepherd (GSMH), un hôpital rural, sur leurs attitudes et leurs connaissances de la circoncision d'enfants pour les raisons médicales. Les participantes s'y connaissaient sur les avantages de la circoncision médicale pour la santé, quoique les connaissances des risques et des avantages de la circoncision d'enfants mâles comparés à la circoncision adulte fussent faibles. Toutes les participantes choisiraient de circoncire leur fils; l’âge préféré pour cela était divers, de l'enfance jusqu’à l'adolescence. Les obstacles principaux cités étaient la douleur et les complications, tandis que les facteurs culturels et religieux étaient cités comme des raisons en faveur ainsi que des raisons contre la circoncision. Nombreux sont les membres de la famille impliqués dans la décision de circoncire un enfant. L'acceptabilité de la circoncision médicale était forte parmi ces participantes mais les préoccupations de la sécurité, la douleur, l'autonomie et les facteurs culturels réduisent l'acceptabilité de la circoncision d'enfants mâles spécifiquement. Il sera donc impératif de fournir des informations sur la circoncision précises et sensibles à la culture du Swaziland pour les mères, les pères et les grands-parents en utilisant les services hôpitaux et communautaires déjà fournis à l'hôpital de GSMH et partout dans le Swaziland. Lorsque cela est possible la circoncision masculine médicale devrait être disponible pour les hommes de tous âges afin que les familles et les jeunes hommes puissent choisir l’âge le plus favourable pour la circoncision.

## 1. Introduction

The number of people in the world living with human immunodeficiency virus (HIV) is higher today than it has ever been, at an estimated 34 million (Joint United Nations Program on HIV and AIDS 2011). In Swaziland, 180,000 people are infected with HIV accounting for just over a quarter of the adult population, which makes Swaziland the country with the highest prevalence of HIV in the world (World Health Organization [WHO] 2011a, 2011b). Prevention of transmission of HIV is therefore a crucial area for development in Swaziland. Medical male circumcision (MMC) reduces the risk of contracting HIV through heterosexual sex by around 60%, as recently demonstrated by three separate randomised controlled trials in southern and eastern Africa (WHO 2007).

As a result, the World Health Organization has identified 13 priority countries in sub-Saharan Africa – those with generalised or hyper-endemic HIV epidemics with a low prevalence of circumcision – for scale up of medical circumcision programmes (WHO 2011a, 2011b). This includes Swaziland, where the prevalence of circumcision is one of the lowest in the region at an average of 8% as there is no cultural tradition of circumcision in Swaziland. According to modelling performed by the United States Agency for International Development, scaling up MMC in Swaziland to 80% of adult males and newborns by 2015 would reduce the HIV incidence by 70% by 2025, preventing 64,000 new infections and save US$332 million in costs for antiretroviral therapy and other HIV-related treatment costs (United States Agency for International Development 2009).

As such, in 2009, the Government of Swaziland introduced a five-year strategy for scale up of MMC with the aim of reaching 80%, or 144,688 males (including 33,000 neonates), as part of a minimum package of services including HIV counselling and testing and safe sex education (Swaziland Male Circumcision Task Force 2009). In collaboration with Population Services International and the Ministry of Health, Good Shepherd Mission Hospital (GSMH) is developing the first integrated comprehensive MMC service for HIV prevention in Swaziland and will be available for males of all ages, including early infants.

Early infant male circumcision (EIMC) is medical circumcision in the first eight weeks of life to reduce the future risk of acquiring HIV and other STIs after sexual debut (rather than any immediate benefit). It has several advantages over adult circumcision such as faster healing, fewer complications and lower cost (WHO 2010). As the Government of Swaziland is moving towards implementing EIMC, it is important to understand the knowledge, motivation and willingness of parents to circumcise their sons. Current evidence from elsewhere in Africa suggests that EIMC is generally very acceptable to parents and grandparents (Mugwanya, Whalen, Celum, Nakku-Joloba, Katabira & Baeten 2011; Plank, Makhema, Kebaabetswe, Hussein, Lesetedi, Halperin, *et al.* 2010; Waters, Stringer, Mugisa, Temba, Bowa & Linyama 2011) with the main concerns identified being pain, loss of cultural identity and complications. However, these studies focus largely on urban inhabitants and, having been conducted in Uganda, Botswana and Zambia, respectively, where some ethnic groups have a long tradition of circumcision, includes populations in which being circumcised or not is an integral part of tribal identity. Within Swaziland, one abstract was identified which found that three-quarters of the men studied would be willing to have a son circumcised (Tsela & Halperin 2006), but the reasons behind this were not discussed. Therefore, there is a clear lack of information on the knowledge and acceptability of EIMC in rural African communities, those that have no ethnic associations with circumcision and in Swaziland specifically, which might inform the service at GSMH. Such information is key at a time when EIMC is being implemented across the country.

## 2. Methods

### 2.1. Study site

GSMH is located near the eastern border of Swaziland, in Siteki, Lubombo region. It is a 201-bed hospital run by the Catholic Diocese and the Government of Swaziland, providing inpatient and outpatient care for the predominantly rural population of the Lubombo region, approximately 210,000 people, as well as specialist HIV, TB and eye services.

### 2.2. Recruitment

This study adopted a qualitative research approach, using semi-structured interviews. Participants were selected using a convenience method, through approaching patients who attended the general outpatients department of the hospital and inviting them to participate. The inclusion criteria were women between the ages of 18–49, not pregnant and well enough to be interviewed for 30 minutes. Nursing staff, on triaging, directed eligible patients to the researcher, who explained the purpose of the research and what participation would involve with the aid of an interpreter, and gave the patient an information sheet in siSwati. Participants then went back into the waiting room to read the information carefully and decide if they want to participate. If the patient decided to participate, she came back into the clinic room, her understanding of the research was checked and she was allowed to ask questions. Written consent was taken using a form translated into siSwati.

A total of 14 women were interviewed in May 2012, ranging in age from 18 to 44. Thirteen were Swazi and one was Mozambican, now living in Swaziland and all described themselves as Christian. Data saturation was achieved at interview 12, but additional two interviews were conducted to ensure no new content was raised.

### 2.3. Interviews

An interview schedule was created through triangulation of the following: existing knowledge of views and behaviour around MMC from a review of the literature; models of behaviour change in particular the Theory of Planned Behaviour (Ajzen 1985) which incorporate perceptions of the risk and benefits of the behaviour as well as social and cultural norms and the idea of self-efficacy to understand health behaviours; and a deductive approach whereby more specific ideas raised by participants were added into the schedule as they arose. Topics included: existing knowledge of MMC; acceptability of medical circumcision (both generally and infant) in the community; willingness to have a son circumcised; and barriers and facilitators to medical circumcision including religious and cultural beliefs. The interview schedule was piloted and continued to develop as participants raised new issues that were incorporated into later interviews. The interview was also used as an opportunity for education and a leaflet in siSwati about the risks and benefits of medical infant circumcision was given at the end with the chance for further discussion.

Interviews were conducted in siSwati using a local interpreter identified by the hospital. The interpreter was trained by the interviewer before the research commenced. Interviews lasted between 20 and 35 minutes and were recorded on an audio device.

### 2.4. Analysis

Thematic analysis was adopted, with phrases or sentences from the first interview being coded into descriptive themes and these themes were used to code further interviews. New codes also came out in later interviews and were incorporated. These more detailed themes were then categorised into a smaller number of broad categories. Coding on the first interview was reviewed by a colleague to ensure validity through a credibility check. Ethics approval for this study was granted by the University of Leeds and Swaziland Ethics Committees.

## 3. Results

Participants had good knowledge of medical circumcision, most mentioning that it is the removal of the foreskin and confers protection against HIV and other STIs. Indeed this was considered the major benefit of the procedure alongside reduced risk of penile cancer and improved genital hygiene. A number of women volunteered that medical circumcision only confers partial protection against HIV.

### 3.1. Timing of male circumcision

All participants were willing to have their son circumcised but there was significant variation in the age at which they felt it would be most appropriate. Autonomy of the son was frequently mentioned as a reason to wait until adolescence whilst some women felt that on growing up, a son would blame his mother for having him circumcised, which he now regrets. One participant made the counter-argument that an uncircumcised son might grow up asking questions about why his mother had not chosen to protect him through medical circumcision, especially if he becomes infected with HIV:

Maybe he can take the decision, maybe at the age of twelve, but maybe sometimes the baby can get infected and he can blame the mother, asking the mother 'why didn't you circumcise me while I was still young?' (010)

Several women felt it would also be important for a boy to understand the reasons why circumcision is necessary and to benefit from the pre-operative counselling and testing.

Some argued that adolescence is a good time for MMC because it is when a boy becomes sexually active. On the other hand, some felt that medical circumcision was best done during childhood in order to protect their son before sexual debut, especially as it may be difficult for a son to tell his mother when he has started having sex and therefore when he should be getting circumcised. Those who preferred medical circumcision at a younger age usually felt the first six months to one year of life to be the best time. It was felt that circumcising at a young age is an opportunity to have a son circumcised before he develops an awareness of the pain and complications.

When this child is older, like my brothers they are refusing now, so you can't take them, you can't force them to do it without them deciding if they do want to go. Whereas when this child is still young, you can do it, just for the sake of his life. (004)

Another commonly stated point was that by circumcising a baby, he will not have any self-consciousness about being circumcised and he will not suffer any discrimination from other children. Early infancy was suggested as being best, by one participant, because babies are less active and not playing with other children which may disrupt the healing process. It was suggested that a benefit of infant circumcision was that it would encourage women to give birth in the hospital, where all the services could be provided in one place:

What they encourage them is that you don't have to deliver at home but at the hospital so you can get all the services they do to the newborn baby. (002)

### 3.2. Fear of complications

Although all the participants would like their son to be circumcised, one of the major factors against it was fear of complications and pain. Excessive bleeding was a particular concern for infants, who were perceived to be at higher risk of death as a result, although it was acknowledged that the likelihood of death from medical circumcision is very remote. In addition, some participants mentioned worries about scarring or injury that would lead to sexual dysfunction in later life:

They say that sometimes they cut at the wrong place so you get scarred for life, injured for life and then you have problems using your penis. They are supposed to cut the foreskin right, so people say that sometimes they cut the tip of the penis and then maybe you have erection problems. (009)

Further to this, one woman expressed concern that medical circumcision was equivalent to castration, resulting in infertility and that by having her one child and son circumcised she would be risking her only chance to have grandchildren:

She has heard people talking that when you are circumcised you get castrated, you don't have children when you get old[er] … What worries her is 'what if I circumcise my one and only child and he doesn't get any children' and she doesn't want to take the blame and say 'I took the decision for my baby'. (012)

On reading the information leaflet, however, many participants became more positive about infant medical circumcision as they learned that it was generally safer and easier than circumcising an adult, that stitches are not used in infants and that local anaesthesia is used to reduce the pain:

I didn't know that they first inject the baby and then they don't stitch the baby, I was afraid of the stitches. (006)

There was some contention over whether the wound would heal more or less quickly in infancy and whether the procedure would be more or less painful. Some felt that the baby being in pain would be difficult for the mother, partly as witness to his crying but also because it would be difficult to tell why he was crying, due to pain or for another reason. In addition, it was suggested that healing was a concern in infancy, due to difficulty in keeping the wound dry:

A newborn is always wearing a nappy so she thinks that the wound cannot easily heal because the diaper gets wet. (013)

Others felt that it would be more painful in adulthood and that an awareness of the likelihood of pain would deter an older male from being circumcised all together. In particular, as erections would be very painful, it is better to circumcise an infant when this particular discomfort would not be a problem:

I think circumcising a baby is good because adults sometimes watch TV, and the penis erects and it gets painful, and a young boy doesn't know anything, it just sleeps. (006)

### 3.3. Religion and culture

Religious and cultural reasons were cited by both those for and against medical circumcision. The lack of a circumcision tradition in Swaziland was suggested as a barrier:

They believe that it is not cultural, not Swazi culture, but for other countries like Mozambique. They don't see the need for Swazis to be circumcised. (003)

Some particular cultural beliefs were raised about circumcision in Swaziland. Some believe that ‘with the foreskin they usually make burning spice after they have removed it’ (010) which discourages some men. This idea has developed in Swaziland due to a local television programme mentioning this as a barrier to a male character being circumcised and has been widely adopted as a cultural belief.

As a result of a recent campaign taking school boys to be circumcised, another woman mentioned a particular fear raised by her children who, having grown up in very rural areas without seeing motorised transport, were afraid of being kidnapped by the bus collecting children to be taken for medical circumcision:

Some children are told about circumcisions at school and they are told there is going to be free transport when they go, so what worries them most is what if the transport doesn't return them, it takes them away for good, maybe for ritual purposes, so they are afraid to do it. (012)

Christianity is the most common religion in Swaziland, and Christian beliefs are firmly embedded in the society. Some considered it ungodly to circumcise and to change the way their bodies were created. One woman mentioned that because Jesus was not circumcised, it is not religiously appropriate. However, this was contradicted by others who believe that Jesus was circumcised and therefore that medical circumcision is acceptable in the church.

### 3.4. Importance of family

The role of different family members appears to be important in the decision to circumcise a son in Swaziland. It was common for women to have to consult the father of the child who would make the final decision. The father's decision may depend on whether the father himself was circumcised. Sometimes, the mother was described as not having much power in the decision, with the father and grandparents having the final say. Others suggested that the decision is a collaborative one, related to the importance of family in Swazi culture:

A child in a family doesn't just belong to one in Swaziland, when you are talking about someone's life everybody has to participate. (006)

### 3.5. Access to services

The distance from people's homes to hospital services was seen as a barrier to some. One woman felt that opening a service at Good Shepherd Hospital would help as the facilities where medical circumcision is available are too far away. Another participant felt that providing medical circumcision in the community setting would be preferable to making people travel to a hospital:

She thinks that it can be best if the health workers can go into the communities and circumcise them there at schools, maybe at the community, rather than them coming to the hospital as it can cost a lot of money and transport. (013)

## 4. Discussion

Knowledge and acceptability of MMC in this study was high, in line with studies conducted in other sub-Saharan African countries (Albert, Akol, L'Engle, Tolley, Ramirez, Opio *et al.* 2011; Bailey, Muga, Poulussen & Abicht 2002; Kebaabetswe, Lockman, Mogwe, Mandevu, Thior, Essex 2003), and may demonstrate the effectiveness of the national campaign for adult medical circumcision, which could be used to address the confusion over the benefits and disadvantages of early infant circumcision amongst women in this region. Many remain fearful about pain and complications, and although this can be partly put down to natural maternal concern, it is clear that accurate information about infant circumcision for medical purposes has not been widely disseminated in this part of Swaziland and that such information would be influential in family decision-making. For example, participants worried about the pain of stitches in a small baby, but when informed that these are not required in the infant procedure and that local anaesthetic is used to reduce the pain, they became more open to it. Infant circumcision is also acceptable in the broader community but this study uncovers some particular cultural beliefs in Swazi communities that might impact upon the decision to circumcise, for example, that circumcision results in infertility; that the foreskin is used to make a spice or the fear of kidnapping when using transport. It will be important to address these specific concerns in educational materials and to provide a high-quality and demonstrably safe service in order to allay fears of complications. Although one participant mentioned the lack of traditional circumcision in Swaziland as a deterrent, the high degree of acceptability and openness to male circumcision in this study could be a product of that lack of tradition and associated ideas of identity, suggesting that with clear and accurate information, adult and infant MMC could be a very effective tool for HIV prevention in Swaziland.

The preferred age varied considerably. This is similar to previous work on the acceptability of neonatal circumcision where a majority of participants would circumcise a son but many would wait until adolescence (Mugwanya *et al.* 2011; Plank *et al.* 2010; Waters *et al.* 2011). With the preferred age ranging from neonates, through toddlers and older children to adolescents, this study highlights the need for flexible services that provide medical circumcision at all ages rather than only at discreet points in a male's life. The strength of this study is its focus on rural inhabitants, which make up 75% of the Swazi population (WHO 2011a, 2011b). GSMH serves a population living in very remote areas that, as suggested by one participant, may benefit most from outreach or community-based services.

This study also shows the importance of all family members in the decision to circumcise a son in Swaziland, particularly fathers. As the circumcision status of fathers can affect the parents’ willingness to circumcise a son, this study suggests that adult medical circumcision campaigns in Swaziland may have the added benefit of increasing uptake of infant circumcision.

There are multiple theories explaining the factors that contribute to a person's decision to enact a certain health behaviour such as EIMC, for example, the Health Beliefs Model (Rosenstock 1966) that synthesises the perceived severity and susceptibility of an illness, and the benefits and barriers of preventive behaviour, to predict a health behaviour. The Theory of Planned Behaviour (Ajzen 1985) goes further in considering social and cultural norms in a person's decision, bringing together attitudes towards a behaviour (knowledge, perceived risks and benefits), subjective norms (perceived societal pressure and expectations from friends and family) and perceived control (self-efficacy) to impact upon the intention to perform a behaviour. In this study the attitudes towards the behaviour were generally positive, particularly after receiving additional information on EIMC, as the perceived susceptibility and severity of HIV were high and the benefits of EIMC were well understood. Subjective norms and perceived control were more variable depending on the participant's interpretation of their religion and the influence of their husbands and family, and their view over whether their son should have autonomy over the decision himself. In light of this, the results of this study suggest that with improved knowledge of the risks and benefits, the most important barriers to mothers considering EIMC will be the views of their husbands and family, and the perceived stance of their church on circumcision. Overall this study identifies barriers to EIMC in this community as pain; fear of complications; autonomy of the son; concern about the use of the foreskin; abduction of children for ritual purposes; influence of the father and extended family, and religion. Facilitators equally included religion as well as HIV and STI prevention; reduced pain and complications in infants; protection before sexual debut and ability for the parents to make the decision in the best interest of their child.

This study goes some way in addressing the lack of information on the views of women around MMC raised by Peltzer, Niang, Muula, Bowa, Okeke, Boiro, *et al.* (2007) and reiterates the need for qualitative research into knowledge and attitudes around infant medical circumcision across sub-Saharan Africa, because although acceptability of medical circumcision is high, local beliefs will vary and will need to be addressed differently according to the context. This will be a crucial aspect of achieving the full potential of MMC to reduce the incidence of HIV.

There are several limitations to this study. The sample size was small and, although data saturation was reached, cannot be considered representative of women in the local region or Swaziland as a whole. Furthermore, as they were recruited in the hospital, it is possible that these women were of a slightly higher socioeconomic background than average for the region as they were able to pay hospital and transport fees. The use of a translator compromises the quality of the data as some of the detail may be lost and it is harder to build a rapport with the participant that would be conducive to more truthful responses. It is possible that the researcher being foreign and seemingly in a position of authority would lead participants to answer in a way that they feel is desired or expected from the researcher. It can also be considered a limitation that only women of childbearing age were recruited. Views of men and grandparents are also important, as born out from the interviews presented here, and merit further investigation.

## 5. Conclusion

EIMC provides an opportunity to dramatically reduce the incidence of HIV in a safe and cost-effective way (WHO 2010). Providing equitable and high-quality services that are designed in response to local needs and beliefs will be essential for it to succeed. This study is the first detailed view of opinions in Swaziland to EIMC, uncovering the main barriers and facilitators to the procedure. Many women living in this rural part of Swaziland find it acceptable, whilst others would prefer to wait until adolescence to circumcise a son. This study also reports good knowledge of circumcision generally although specific knowledge of the risks and benefits of infant circumcision is poor. Therefore, detailed information and culturally sensitive education materials on early infant circumcision should be distributed. Existing services such as antenatal clinics and maternity wards provide an opportunity to reach mothers. Men are particularly influential in the decision and should be targeted, perhaps through their own interaction with medical circumcision and reproductive health services. Specifically, community outreach such as the home-based care team at GSMH can be used to reach multiple generations of families and those who do not regularly access hospital services. Services available to boys of all ages will provide the best opportunity to achieve high uptake.
